# Biomimetic Strategies for Subsurface Enamel Remineralization: Depth-Dependent Effects of Biomimetic Agents Compared with Fluoride

**DOI:** 10.3390/biomimetics11080525

**Published:** 2026-07-24

**Authors:** İlknur Akay Dede, Suat Özcan

**Affiliations:** Department of Restorative Dentistry, Faculty of Dentistry, University of Gazi, Ankara 06490, Turkey

**Keywords:** dental enamel, tooth remineralization, hydroxyapatites, caseins, fluorides

## Abstract

This study compared the remineralization potential of three commercially available fluoride-free biomimetic formulations containing nanohydroxyapatite (nHAp), casein phosphopeptide–amorphous calcium phosphate (CPP-ACP), and bioactive glass (BAG) with a conventional 1450 ppm fluoride-containing dentifrice using cross-sectional microhardness and SEM analyses. Seventy extracted human mandibular molars were randomly allocated to five groups (*n* = 14). Artificial carious lesions were created by 72 h of demineralization followed by a 14-day pH-cycling regimen with twice-daily treatment applications. Cross-sectional microhardness was measured at depths of 30, 50, 100, and 150 µm, and SEM was used to evaluate morphological changes. Data were analyzed using one-way ANOVA with appropriate post hoc tests (Tukey’s HSD or Tamhane’s T2, depending on variance homogeneity) and repeated-measures ANOVA to assess depth-dependent differences among groups (α = 0.05). Demineralization significantly reduced microhardness at all depths (*p* < 0.05). At 30 µm, the nHAp, CPP-ACP, and fluoride formulations showed similar remineralization, whereas the BAG-containing formulation exhibited lower superficial recovery. At 100–150 µm, the nHAp formulation demonstrated the greatest recovery, followed by the CPP-ACP and BAG formulations, while the fluoride dentifrice showed progressively lower recovery. A significant depth-by-group interaction confirmed distinct depth-dependent remineralization profiles. Within the limitations of this in vitro study, all tested formulations promoted enamel remineralization but exhibited different depth-dependent recovery patterns.

## 1. Introduction

Dental caries is a dynamic, multifactorial disease that results from interactions among tooth structure, oral microbial composition, and environmental factors such as diet and saliva, and is characterized by cycles of demineralization and remineralization at the tooth surface [1,2]. The development of the disease is influenced by factors such as sugar intake, inadequate oral hygiene, and individual host conditions [3]. The caries process begins with subclinical caries lesions, progressing to the formation of white spot lesions (WSLs), which may develop into cavitation at the enamel surface or extend into dentin. WSLs are characterized by loss of enamel translucency due to subsurface porosity, which alters the refractive index and results in increased light scattering and reduced fluorescence. Clinically, they appear as opaque white areas on the enamel surface, while subclinically they represent mineral loss beneath a relatively intact surface layer. These lesions are reversible, as the relatively intact surface layer and porous mineral-deficient structure allow diffusion of calcium and phosphate ions into the subsurface enamel, enabling mineral redeposition [4,5].

Remineralization is the process of mineral recovery in demineralized dental tissues through the re-deposition of calcium and phosphate ions into the crystal structure, forming hydroxyapatite, fluorhydroxyapatite, or carbonated hydroxyapatite, depending on the ionic environment. This process occurs when the environmental pH exceeds the critical pH of the respective tooth mineral, approximately 5.5 for enamel hydroxyapatite, 6.5 for dentin, and 4.5 for fluorapatite [6,7,8]. The progression or reversal of lesions depends on the balance between pathological factors, such as cariogenic bacteria, frequent exposure to fermentable carbohydrates, and impaired salivary function, and protective factors, including adequate saliva, fluoride availability, and remineralizing ions [9,10,11]. Early enamel caries lesions are defined as non-cavitated lesions characterized by visible changes in enamel color and reduced mineral density compared with adjacent sound enamel [12]. Therefore, non-restorative management of early caries lesions has become a key objective of minimally invasive dentistry [13].

Fluoride has long been used as an effective agent for caries prevention and enhancement of remineralization [14]. By interacting with calcium and phosphate ions, fluoride promotes the formation of less soluble fluoridated apatite phases [5]. However, remineralization induced by fluoride is often concentrated in the outer enamel layer, where fluorapatite and CaF_2_-like precipitates may form, potentially limiting ion diffusion into deeper regions of the lesion [15,16,17]. This depth-dependent remineralization pattern has prompted increasing interest in biomimetic materials capable of delivering calcium and phosphate ions directly into deeper regions of the lesion.

To overcome these limitations, biomimetic agents such as nHAp, CPP-ACP, and BAG have been developed. These materials provide bioavailable calcium and phosphate ions and may support mineral deposition both at the surface and within subsurface lesion regions [5].

Due to its structural similarity to enamel apatite, nHAp may integrate into enamel defects and act as a source of calcium and phosphate ions, facilitating mineral deposition within subsurface lesions [18,19,20,21]. It has also been reported to adsorb to enamel surfaces and fill interprismatic spaces, contributing to its remineralizing potential [22].

CPP-ACP consists of casein-derived phosphopeptides that stabilize calcium and phosphate ions, maintaining them in a bioavailable form and promoting their diffusion into subsurface enamel lesions, particularly under acidic conditions [15,23,24,25,26]. In the presence of fluoride, CPP facilitates the formation of amorphous calcium fluoride phosphate (ACFP), further enhancing remineralization [15].

Bioactive glasses react with saliva to form a carbonated hydroxyapatite layer and release calcium and phosphate ions, thereby supporting remineralization within subsurface lesions [26,27]. BAG also exhibits antibacterial properties, primarily demonstrated in in vitro studies, by releasing alkaline ions and increasing the local pH, thereby inhibiting biofilm formation [28]. The addition of elements such as silver, magnesium, zinc, and strontium can further enhance these effects [29].

Microhardness testing is widely used to assess changes in dental hard tissues, providing indirect evidence of demineralization and remineralization through changes in enamel hardness. Cross-sectional microhardness analysis enables depth-specific quantification of remineralization and provides insight into mineral distribution gradients within the lesion body [30]. Additionally, scanning electron microscopy (SEM) provides detailed visualization of surface morphology and allows qualitative evaluation of structural changes after treatment [31].

A variety of agents have been investigated for their remineralization potential on early enamel lesions. However, variations in lesion models, pH-cycling protocols, and evaluation methods across studies may influence the reported outcomes and make direct comparison difficult. Consistent with this, recent systematic reviews have reported considerable heterogeneity among studies, which may limit the comparability of findings and complicate the interpretation of results [26]. While numerous studies have investigated the remineralization potential of fluoride and biomimetic agents individually, comparative data evaluating remineralization at multiple defined subsurface levels under identical experimental conditions remain limited. In addition, although SEM is commonly used to examine enamel morphology, many studies primarily focus on surface features, while cross-sectional evaluation of subsurface changes is not always included. Therefore, this in vitro study aimed to compare the remineralization potential of commercially available fluoride-free biomimetic formulations and a conventional fluoride-containing dentifrice at multiple defined subsurface depths using cross-sectional microhardness and SEM analyses. The null hypothesis was that the fluoride-free formulations would show no significant difference in remineralization efficacy at any tested subsurface depth compared with the 1450 ppm fluoride-containing dentifrice.

## 2. Materials and Methods

This in vitro study investigating the remineralization potential of different commercially available biomimetic formulations and a conventional fluoride-containing dentifrice on artificially demineralized enamel was approved by the Clinical Research Ethics Committee of Gazi University Faculty of Dentistry (Approval No. E-21071282-050.99-711205; Approval Date: 27 July 2023).

### 2.1. Sample Selection and Preparation

A total of 70 sound, caries-free, unrestored human permanent mandibular first and second molar teeth extracted for periodontal reasons within the last month were used to provide enamel specimens in this study. After extraction, the teeth were rinsed with distilled water and mechanically cleaned using periodontal curettes to remove soft tissue remnants, followed by polishing with fluoride-free pumice and brush, and rinsed under running water [32]. The specimens were then stored in distilled water at 37 °C for up to one month prior to specimen preparation, with daily renewal of the storage solution. A power analysis was performed using G*Power software (Version 3.1.9.6) to determine the required sample size. The effect size of 0.50 was estimated based on mean and standard deviation values reported in previously published in vitro studies on enamel remineralization [33]. Using a significance level (α) of 0.05, a statistical power of 80%, and an effect size of 0.50, the minimum required sample size was calculated as 55 specimens (*n* = 11 per group). To account for potential specimen loss during preparation and experimental procedures, 14 specimens were allocated to each group.

The roots were sectioned from the crowns using a diamond separating disc under water cooling. Each crown was then sectioned mesiodistally to obtain buccal and lingual halves. The buccal half was designated as the demineralization and treatment area, and the lingual half was used as the sound control. Each specimen was embedded in 2 × 2 cm molds using cold-cure acrylic resin. Specimens were randomly assigned to the experimental groups using a computer-generated randomization sequence generated with the RAND function in Microsoft Excel.

Three 3 × 3 mm windows were prepared: two on the buccal surface and one on the lingual surface. Adhesive tape was used to define the exposed areas, and the remaining surfaces were coated with two layers of acid-resistant nail varnish to prevent solution penetration during pH cycling. Of the two buccal windows, one was designated for demineralization only, and the other for treatment application. The lingual window served as the sound enamel reference [32]. Following artificial lesion formation, the demineralized-reference buccal window was covered with acid-resistant nail varnish and remained sealed for the remainder of the experiment, whereas the adjacent buccal window remained exposed for treatment application and pH cycling. The enamel surfaces were not flattened, and all windows were prepared following the natural curvature of the tooth surface. The window dimensions (3 × 3 mm) were standardized across specimens without adjustment for surface curvature. Lingual halves were stored in distilled water at 37 °C until the end of the experiment to preserve their integrity [34].

### 2.2. Artificial Lesion Formation and pH Cycling

The demineralization and remineralization solutions were prepared using analytical-grade chemicals and distilled water, following a protocol similar to that described by Cate et al. [35]. This protocol has been reported to produce subsurface enamel demineralization without surface erosion [36]. Artificial caries lesions were created using a demineralization solution (2.2 mM CaCl_2_, 2.2 mM NaH_2_PO_4_, 0.05 M acetic acid, adjusted to pH 4.4 with 1 M KOH). Buccal windows were immersed for 72 h at 37 °C in an incubator, and the solution was renewed every 24 h. Each specimen was placed individually in a standardized container, and 20 mL of solution was added to ensure complete immersion of the exposed enamel window (3 × 3 mm). The same volume was applied for both demineralization and remineralization solutions throughout the pH cycling regimen.

After initial lesion formation, a 14-day pH cycling regimen was applied. The demineralization solution used during pH cycling was identical in composition to that used for initial artificial lesion formation. The duration of the pH cycling protocol was selected based on previous reports indicating that bioactive glass requires approximately two weeks for ion release and direct hydroxycarbonate apatite (HCA) formation [37], and that CPP-ACP systems demonstrate effective remineralization of early enamel lesions within a similar time frame under dynamic pH conditions [38]. The nominal active components of the tested formulations differ in their reported ion-release characteristics: BAG releases calcium, phosphate, sodium, and silicate ions; nHAp provides calcium and phosphate ions; and CPP-ACP stabilizes and delivers bioavailable calcium and phosphate ions [13].

The daily pH cycling schedule was performed at 37 °C following a protocol similar to that described by Kumar et al., with specimens immersed in the demineralization solution for 6 h and in the remineralization solution (1.5 mM CaCl_2_, 0.9 mM NaH_2_PO_4_, 0.15 M KCl, pH 7.0, prepared without the addition of a buffering agent) for approximately 16 h per day [39]. Both demineralization and remineralization solutions were renewed daily throughout the pH-cycling period to ensure chemical stability and consistency of the experimental conditions. The remaining 2-h period of each 24-h cycle was allocated to rinsing and treatment application procedures. During this interval, the specimens were removed from the solutions, rinsed with distilled water, and then treated individually with the respective formulations for 3 min, twice daily (before and after the demineralization phase), consistent with the recommended twice-daily use of the tested dentifrices. The 3-min contact time was selected based on the manufacturer’s recommended minimum contact time for CPP-ACP (GC Tooth Mousse) [40] and the same duration was applied uniformly to all groups to allow direct comparison under identical conditions. This protocol is also consistent with previously published in vitro remineralization studies [32,41]. The specimens were kept in distilled water between and after applications to ensure equal total exposure time for all specimens. Throughout the 14-day pH-cycling period, the demineralized-reference buccal window remained sealed with nail varnish and was not exposed to either the pH-cycling solutions or the tested formulations. In contrast, the adjacent buccal window remained exposed and received the assigned treatment protocol.

No dentifrice slurry was prepared; instead, the tested materials were applied directly in their original, undiluted form using a microbrush to cover the 3 × 3 mm treatment window with an approximate thickness of 1 mm. This approach was preferred to maintain consistency across materials with different physical forms (e.g., paste and cream) and to evaluate their intrinsic remineralization potential without dilution effects. After each application, the specimens were rinsed with distilled water and stored in distilled water until the next phase of the experimental cycle. The tested formulations included:

- nHAp-containing dentifrice (Biorepair^®^, Coswell S.p.A., Funo, Bologna, Italy);
- CPP-ACP-containing dentrifice (GC Tooth Mousse, GC Corp., Tokyo, Japan);
- BAG-containing dentifrice (BioMin C, BioMin Technologies Ltd., Stoke-on-Trent, UK);
- 1450 ppm fluoride-containing dentifrice (Colgate Triple Action, Colgate-Palmolive, Guangzhou, China);
- Distilled water (negative control; no dentifrice applied).

A negative control group was included, in which specimens were subjected only to the pH-cycling regimen without the application of any remineralizing agent. A fluoride-containing group (1450 ppm) was used as a positive control due to its well-established remineralization efficacy. The study included fluoride-free biomimetic formulations and a fluoride-containing formulation, and their compositions are summarized in Table 1.

**Figure 1:**
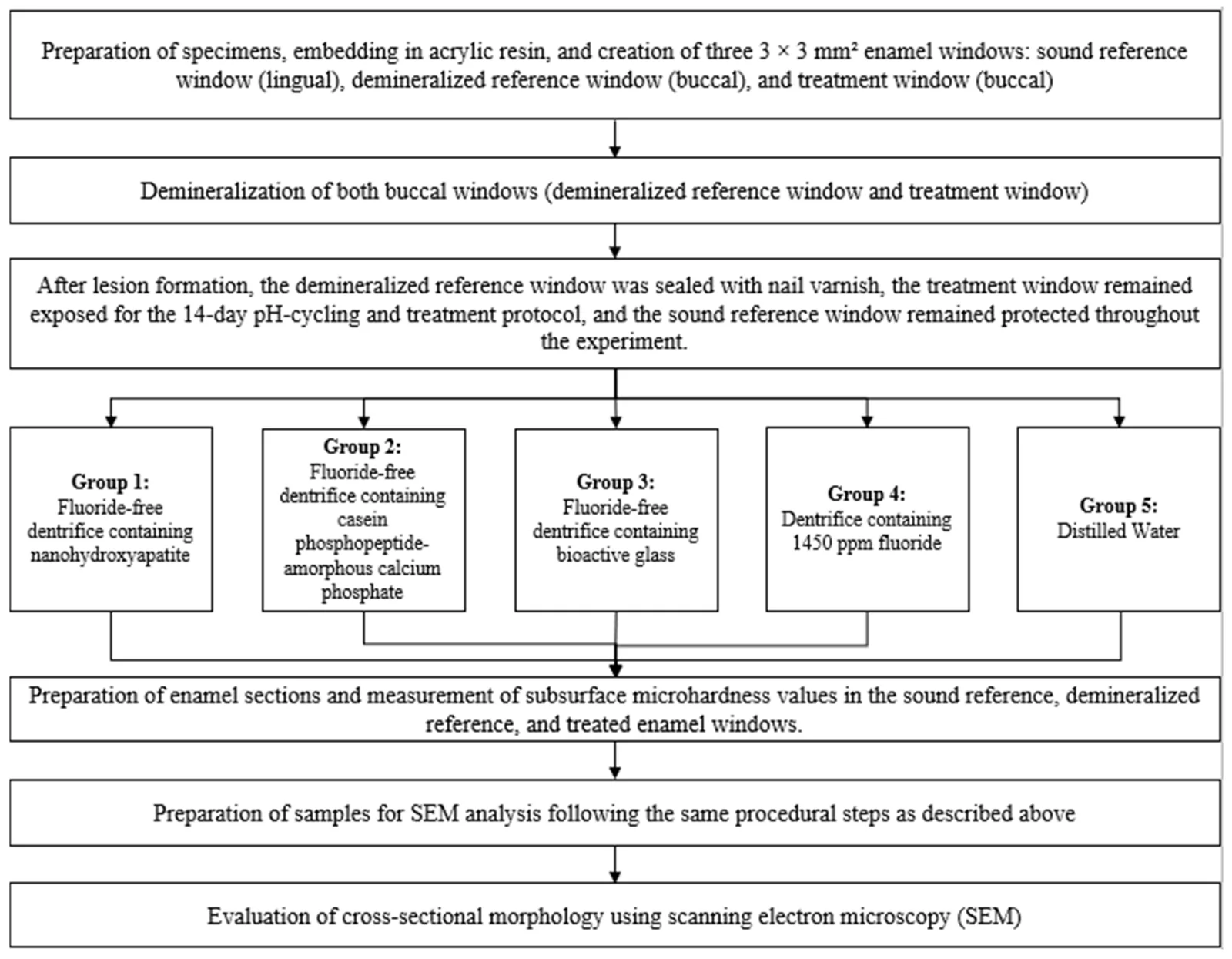
Schematic representation of the experimental design showing sample preparation, demineralization, pH cycling, remineralization treatment, and evaluation steps.

A schematic representation of the experimental design is presented in Figure 1.

### 2.3. Microhardness Measurements

All measurements were performed by a single calibrated operator; however, blinding was not applied. After pH cycling, specimens were sectioned through the center of the 3 × 3 mm buccal and lingual windows in the cervico-occlusal direction using a precision saw (Isomet 4000, Buehler, Lake Bluff, IL, USA). Microhardness measurements were performed using a Vickers hardness tester (Shimadzu HMV-700, Kyoto, Japan) with a 100 g load for 15 s at depths of 30 µm, 50 µm, 100 µm, and 150 µm from the surface, and values were expressed as Vickers hardness numbers (VHN, kg/mm^2^). The Vickers hardness test was selected as it has been reported to be more suitable than the Knoop test for tooth hardness measurements, owing to its consistent square indentation geometry and ease of diagonal measurement [42]. The selected load (100 g) and dwell time (15 s) were determined based on previously described microhardness testing conditions for enamel [43]. The use of a 100 g load enables the formation of clearly measurable indentations, and the selected dwell time is consistent with a previous study indicating minimal influence of dwell time variation on microhardness values [44]. The 30 µm depth was selected based on reports indicating that fluoride-induced remineralization is primarily limited to the superficial enamel layer and may not penetrate beyond approximately 30 µm [9,45]. Similar cross-sectional microhardness studies have evaluated enamel at subsurface depths of 30, 40, 50, and 100 µm [46]. The maximum depth of 150 µm was selected in accordance with a previous study using similar demineralization conditions, which reported lesion depths of approximately 150–200 µm after 96 h of exposure [36]. In the present study, a shorter demineralization period (72 h) was applied. Intermediate depths (50 and 100 µm) were included to evaluate hardness recovery at intermediate subsurface assessment depths and to characterize depth-dependent remineralization behavior.

Three indentations were made at each depth, with a minimum spacing of 150 µm between adjacent indentations to avoid interaction effects, consistent with the recommended minimum distance of 2.5 times the diagonal length [44]. The mean of the three indentations at each depth was calculated and used for subsequent statistical analyses. CSMHR was calculated individually for each specimen at each depth using the matched sound, demineralized, and treated enamel values obtained from the same tooth. The resulting specimen-level CSMHR values were used for statistical analysis. The cross-sectional microhardness recovery (CSMHR) percentage was calculated to determine the degree of remineralization at each depth using the following formula: CSMHR (%) = (VHN_post-pH-cycling_ − VHN_carious_)/(VHN_baseline_ − VHN_carious_) × 100. In this formula, VHN_baseline_ refers to the hardness of sound enamel, VHN_carious_ refers to the hardness after demineralization, and VHN_post-pH-cycling_ refers to the hardness measured after the remineralization treatment. This formula, originally described in early in vitro remineralization studies and subsequently adopted in numerous enamel remineralization investigations, enables quantitative assessment of hardness recovery relative to sound enamel values [30,47]. It represents the ratio between the hardness gain after treatment and the maximum recoverable hardness difference between sound and demineralized enamel.

To enable integrated hardness deficit assessment consistent with previously described methodology [48], the area under the hardness-versus-depth curve was calculated individually for each specimen using the trapezoidal rule over the measured depth range of 30–150 µm. Integration was performed across the 30–50, 50–100, and 100–150 µm intervals. Because no direct hardness measurement was obtained at the enamel surface (0 µm), the 0–30 µm region was not extrapolated and was therefore excluded from the integration. The resulting values were expressed as VHN·µm. The demineralization-associated integrated hardness deficit (ΔS_carious_) was defined as the difference between the integrated hardness area of the matched sound enamel profile (S_baseline_) and that of the demineralized enamel profile (S_demin_). Similarly, the residual integrated hardness deficit after treatment (ΔS_post-pH-cycling_) was calculated as the difference between Sbaseline and the integrated hardness area of the post-treatment enamel profile (_Spost-pH-cycling_). Integrated hardness recovery (ΔΔS) was subsequently calculated as ΔS_carious_ − ΔS_post-pH-cycling_, with higher values indicating greater recovery of the demineralization-associated hardness deficit. The upper integration limit of 150 µm corresponded to the deepest predefined microhardness measurement point, selected on the basis of lesion depths reported for comparable demineralization protocols [36].

### 2.4. SEM Analysis

Two specimens from each group were prepared for morphological analysis. After sectioning, specimens were placed in an ultrasonic bath for 30 min to remove the smear layer and air-dried in a dust-free environment for 48 h. The samples were sputter-coated with gold–palladium using a vacuum coating device (EMITECH K550X; Emitech Ltd., Ashford, UK) and examined under a scanning electron microscope (EVO-40 series; Carl Zeiss, Oberkochen, Germany) at an accelerating voltage of 20 kV. Representative images were obtained at magnifications of ×500, ×1000, and ×2000. The preparation and imaging procedures followed previously described SEM protocols for enamel specimens [31,49]. SEM analysis was performed on cross-sectioned enamel specimens as a qualitative method to assess subsurface morphological characteristics associated with demineralization and remineralization as widely reported in studies on initial caries-like and erosion lesions [50,51,52]. In addition, SEM provides high-resolution visualization of structural changes associated with demineralization and remineralization, supporting the interpretation of microhardness findings [53].

### 2.5. Statistical Analysis

All data obtained from the study were analyzed using IBM SPSS Statistics 22 (IBM SPSS Inc., Chicago, IL, USA). The normality of data distribution was assessed using the Shapiro–Wilk test, and homogeneity of variances was evaluated with Levene’s test.

To evaluate the effect of enamel depth on remineralization and to determine whether depth-dependent recovery profiles differed among the experimental groups, a repeated-measures analysis of variance (repeated-measures ANOVA) was performed with enamel depth (30, 50, 100, and 150 µm) as the within-subject factor and treatment group as the between-subject factor. Mauchly’s test was used to assess the assumption of sphericity. When sphericity was violated, Greenhouse–Geisser corrections were applied.

CSMHR values at each individual depth and integrated hardness parameters (ΔS_carious_, ΔS_post-pH-cycling_, and ΔΔS) were compared among groups using one-way ANOVA. Tukey’s post hoc test was applied when homogeneity of variances was met, whereas Tamhane’s T2 test was used when this assumption was violated. The level of statistical significance was set at *p* < 0.05.

## 3. Results

Cross-sectional microhardness measurements were performed on the buccal halves containing demineralized and treated regions, while the lingual halves served as sound enamel controls. CSMHR values were calculated for each group and statistically compared. Significant differences were observed among the groups at various measurement depths (*p* < 0.001).

### 3.1. Evaluation of Cross-Sectional Microhardness

**Figure 2:**
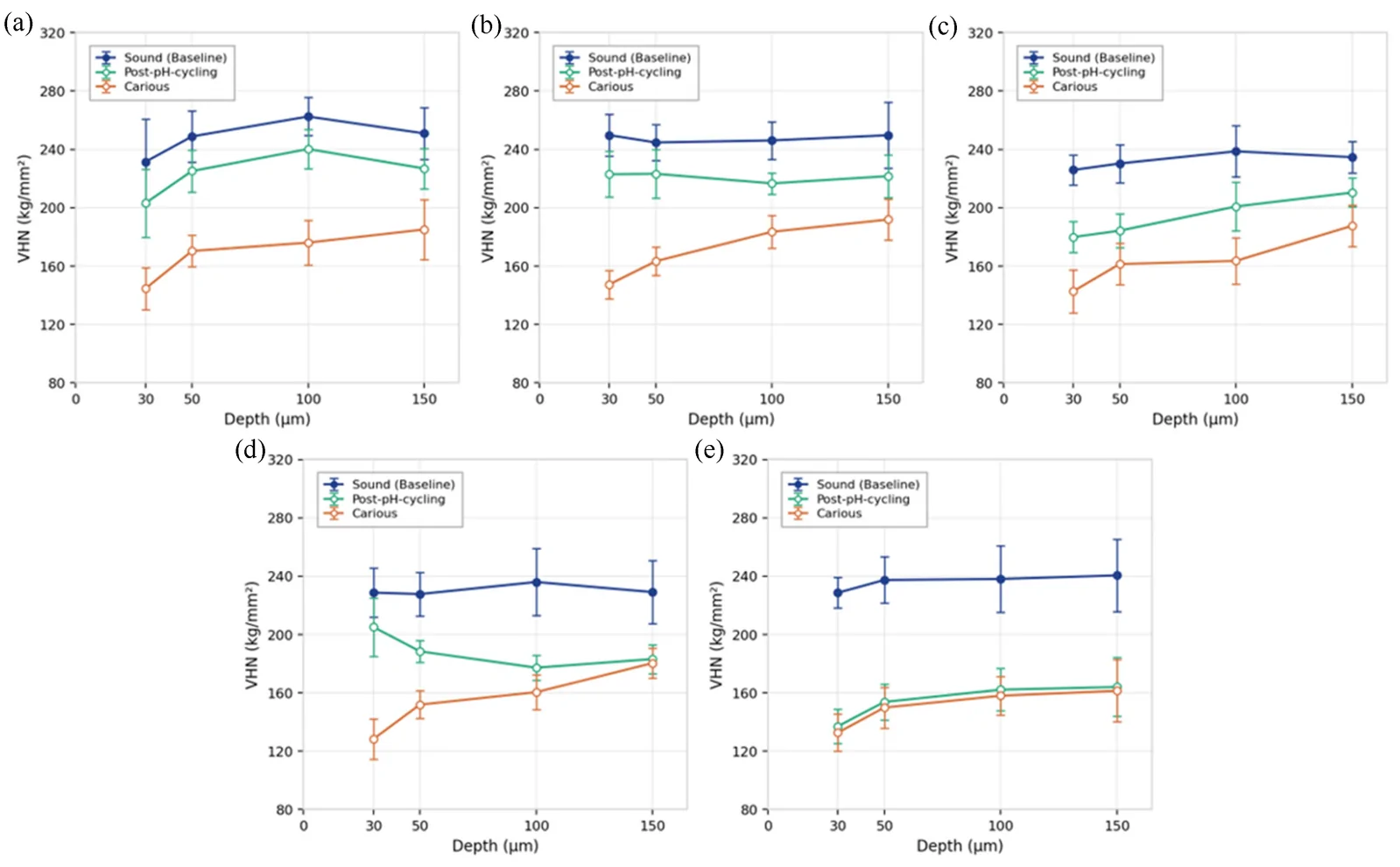
Cross-sectional microhardness (VHN, kg/mm^2^) values of enamel at different depths (30, 50, 100, and 150 µm) for each experimental group. Data are presented as mean ± standard deviation. Error bars indicate standard deviations. (**a**) nHAp, (**b**) CPP-ACP, (**c**) BAG, (**d**) Fluoride, (**e**) Control.

No statistically significant differences were observed among the groups in terms of ΔS_carious_ values (one-way ANOVA, *p* = 0.102), indicating comparable initial lesion severity. Following demineralization, subsurface microhardness values were significantly reduced in all groups compared with sound enamel (*p* < 0.001). After treatment with the tested formulations, a significant increase in microhardness was observed, indicating partial remineralization (*p* < 0.001). Table 2 presents the mean (±SD) Vickers microhardness (VHN, kg/mm^2^) values of baseline (sound), carious (demineralized), and post-pH-cycling (remineralized) enamel at depths of 30, 50, 100, and 150 µm for each group, while the corresponding depth-dependent microhardness profiles are illustrated in Figure 2.

### 3.2. Comparison of Recovery Percentages Among Groups

CSMHR (%) values were calculated to quantify the remineralization efficiency of the tested formulations (Table 3). Significant differences were observed among the groups at all measured depths (one-way ANOVA, *p* < 0.001).

At 30 µm, the control group showed the lowest recovery percentage (4.47 ± 2.54%), significantly lower than all other groups (*p* < 0.001). The BAG formulation demonstrated lower recovery (44.78 ± 5.51%) than the nHAp formulation (67.02 ± 7.35%), CPP–ACP formulation (73.95 ± 12.44%), and fluoride formulation (75.80 ± 11.74%) (*p* < 0.05). No significant differences were observed among the nHAp, CPP–ACP, and fluoride formulations (*p* > 0.05).

At 50 µm, significant differences were observed among the groups (*p* < 0.001). The control group again exhibited the lowest recovery (4.46 ± 2.94%), followed by the BAG formulation (33.05 ± 3.68%), which showed significantly lower recovery than the nHAp, CPP–ACP, and fluoride formulations (*p* < 0.05). The fluoride formulation exhibited lower recovery (48.29 ± 9.66%) than the nHAp formulation (69.31 ± 7.76%) and CPP–ACP formulation (72.63 ± 10.59%) (*p* < 0.05), whereas no significant difference was observed between the nHAp and CPP–ACP formulations (*p* > 0.05).

At 100 µm, significant differences were observed among the groups (*p* < 0.001). The nHAp formulation exhibited the highest recovery (68.31 ± 4.97%), significantly higher than the BAG, CPP–ACP, and fluoride formulations (*p* < 0.05). The control group showed the lowest recovery (5.29 ± 3.44%), whereas the fluoride formulation demonstrated significantly lower recovery than the BAG and CPP–ACP formulations (*p* < 0.05). No significant difference was observed between the BAG and CPP–ACP formulations (*p* > 0.05).

**Figure 3:**
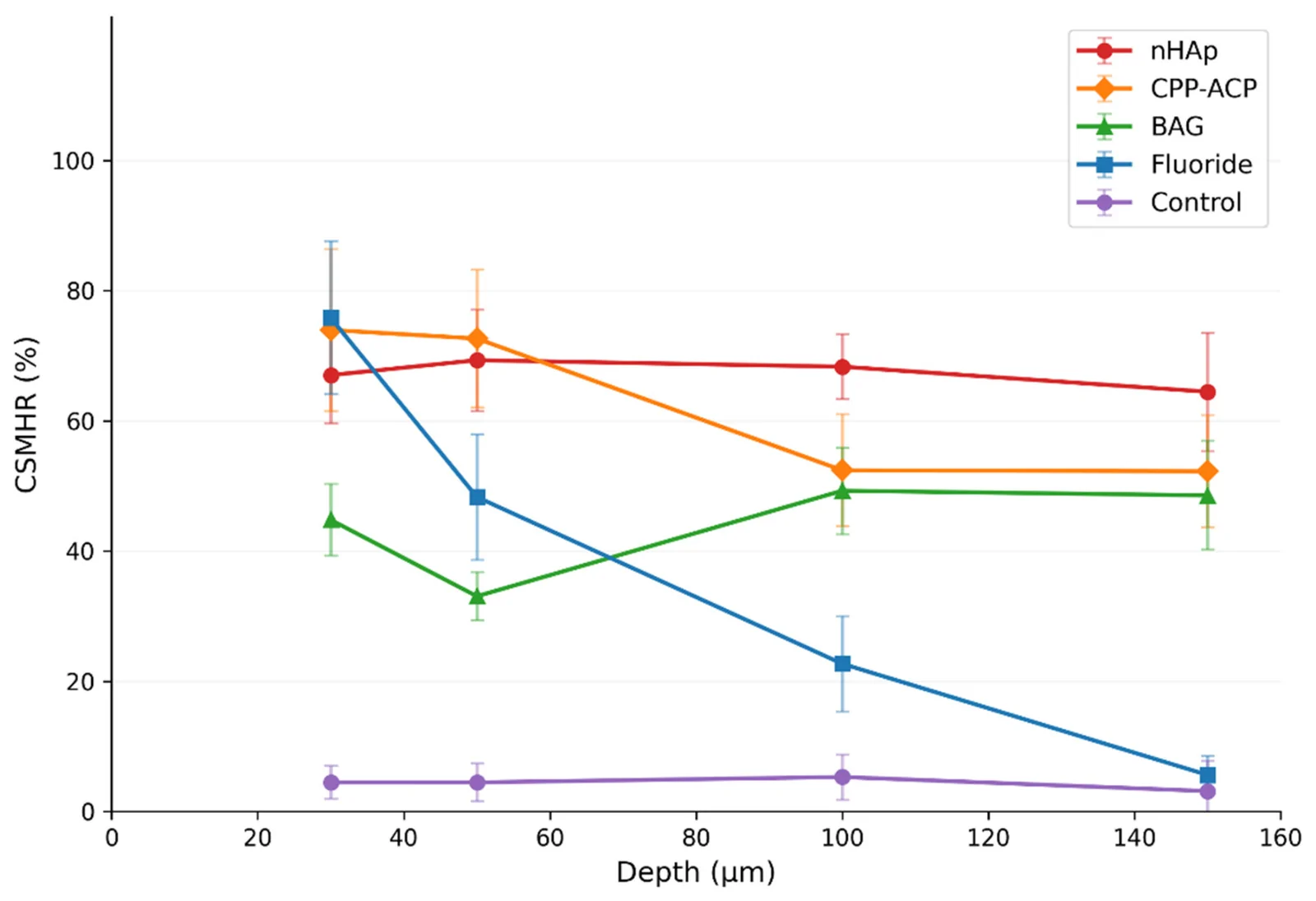
Depth-dependent cross-sectional microhardness recovery (CSMHR, %) across subsurface enamel depths (30, 50, 100, and 150 µm) for all experimental groups (nHAp, CPP-ACP, BAG, Fluoride, and Control). Error bars indicate standard deviation (SD).

At 150 µm, the nHAp formulation again demonstrated the highest recovery (64.46 ± 9.08%), significantly higher than all other groups (*p* < 0.05). The BAG and CPP–ACP formulations showed comparable recovery rates, both significantly higher than the fluoride formulation and the control group (*p* < 0.05). No significant difference was observed between the fluoride formulation and the control group at this depth (*p* > 0.05). These findings are summarized in Table 3 and illustrated in Figure 3.

To evaluate whether treatment effects varied according to enamel depth, a repeated-measures ANOVA was performed with depth as the within-subject factor and group as the between-subject factor. Because Mauchly’s test indicated a violation of sphericity (*p* = 0.024), Greenhouse–Geisser corrections were applied. A significant main effect of depth was observed (F(2.643, 153.318) = 80.979, *p* < 0.001, partial η^2^ = 0.583). A significant depth × group interaction was also observed (F(10.574, 153.318) = 54.160, *p* < 0.001, partial η^2^ = 0.789), indicating that the remineralization profile differed among groups across enamel depths.

ΔΔS (VHN·µm) analysis revealed statistically significant differences among the groups (*p* < 0.001). The nHAp and CPP–ACP formulations exhibited the greatest integrated hardness recovery, with no statistically significant difference between them (*p* > 0.05). Both groups demonstrated significantly higher ΔΔS values than the BAG, fluoride, and control groups (*p* < 0.05). No significant difference was observed between the BAG and fluoride formulations (*p* > 0.05), while the control group showed the lowest values. These findings are summarized in Table 4.

### 3.3. Scanning Electron Microscopy (SEM) Observations

**Figure 4:**
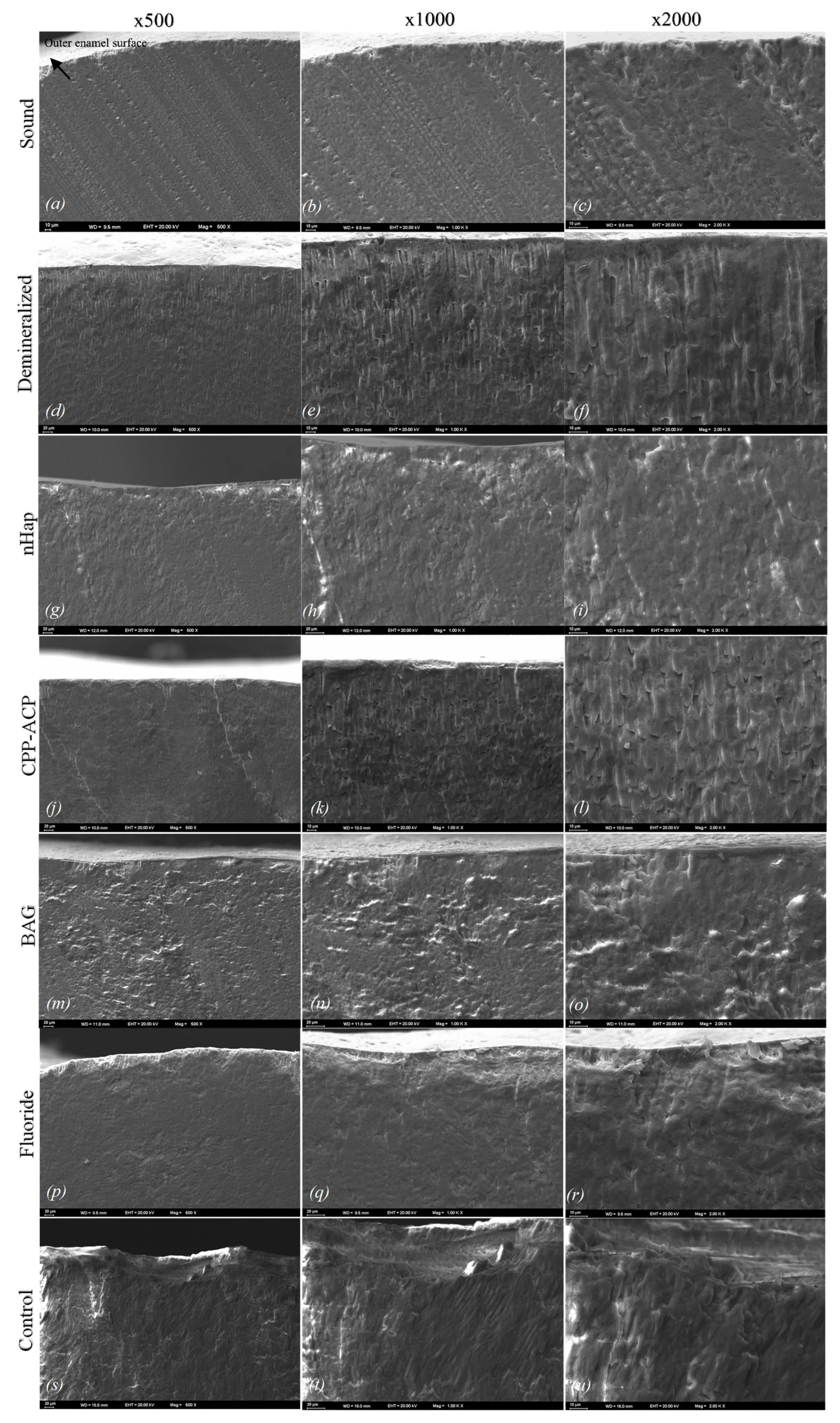
Representative SEM images obtained from the cross-sectional surfaces of sectioned enamel specimens at ×500, ×1000, and ×2000 magnifications. (**a**–**c**) Sound enamel demonstrating a compact and uniform morphology with clearly distinguishable enamel prisms and interprismatic regions. (**d**–**f**) Demineralized enamel characterized by disruption of prism architecture and the presence of surface and subsurface porosities. (**g**–**i**) nHAp-treated specimens showing a relatively uniform surface layer with apparent extension into interprismatic areas. (**j**–**l**) CPP-ACP-treated specimens displaying amorphous-appearing deposits with partial occlusion of porosities and residual voids. (**m**–**o**) BAG-treated specimens presenting irregularly distributed surface deposits and partial coverage of interprismatic spaces. (**p**–**r**) Fluoride-treated specimens exhibiting surface features predominantly confined to the outer enamel region. (**s**–**u**) Demineralized control specimens maintaining porous morphology. The orientation of the cross-section (outer enamel surface indicated by the arrow) is consistent across all panels.

Representative SEM images obtained from the cross-sectional surfaces of sound, demineralized, and treated enamel specimens are shown in Figure 4. Morphological changes observed along the cross-sections are described below. Sound enamel exhibited a smooth and compact surface with clearly identifiable prism and interprismatic structures. Following demineralization, a porous and irregular surface morphology with evident prism dissolution was observed, consistent with enamel demineralization. After remineralization, surface alterations were observed in all treatment groups. The nHAp group showed a relatively continuous surface layer with apparent surface features within interprismatic regions. The CPP-ACP group demonstrated partial occlusion of surface porosities by amorphous-appearing deposits. The BAG group exhibited irregular surface deposits. In contrast, the fluoride group presented surface changes primarily confined to the outer enamel layer.

## 4. Discussion

This in vitro study compared the remineralization potential of commercially available formulations containing nHAp, CPP-ACP, and BAG with a 1450 ppm fluoride-containing dentifrice on artificially demineralized enamel using a 14-day pH-cycling model. All tested formulations produced statistically significant increases in microhardness following treatment, indicating remineralization under the present experimental conditions. However, the extent and depth-distribution of remineralization differed among the formulations, reflecting differences in their observed depth-dependent remineralization profiles.

Over recent decades, clinical and scientific evidence has demonstrated the benefits of minimally invasive dental treatments. One of the main objectives of this concept is the remineralization of initial caries lesions [11]. Fluoride remains the most established non-invasive approach for enhancing enamel resistance through fluorapatite formation [54,55]. Nonetheless, evidence suggests that fluoride primarily targets the outermost enamel layer, with limited penetration beyond approximately 10–30 μm [11,16]. This limited diffusion capacity, particularly in deeper non-cavitated lesions, has led to interest in biomimetic strategies that provide bioavailable calcium and phosphate to support subsurface mineral replacement [4,9]. In light of these considerations, the depth-dependent behavior of the tested formulations warrants careful evaluation.

In this study, the nHAp-containing formulation demonstrated the most consistent hardness recovery across all assessment depths. This finding is consistent with previous reports suggesting that nanometer-scale apatite particles may contribute to remineralization by increasing the local availability of calcium and phosphate ions and by providing nucleation sites for apatite deposition [13,56]. Similar depth-dependent superiority of the nHAp-containing formulation over the fluoride-containing formulation was reported by Tschoppe et al. [57]. Consistent with these findings, Swarup et al. [58] showed that 10% nHAp exhibited significantly higher microhardness recovery and Ca/P ratios than 2% NaF in an in vitro model. Taken together, these observations suggest that the nHAp-containing formulation may support remineralization at multiple subsurface levels within the limitations of the present model. Similar to the results of Tschoppe et al. [57], the nHAp-containing formulation maintained comparatively higher recovery values than the fluoride-containing formulation at increasing depths, although direct extrapolation to clinical lesion behavior should be made with caution.

The CPP-ACP-containing formulation also produced significant remineralization at all depths, with greatest improvement at 30 μm and a gradual reduction at deeper levels. This trend is consistent with the proposed mechanism of CPP-ACP, which has been reported to stabilize calcium and phosphate ions in metastable nanocomplexes [59]. Several studies have reported strong remineralization at both surface and subsurface levels with CPP-ACP [59,60]. However, our results differ to some extent from Vyavhare et al. [41], who found that CPP-ACP provided lower remineralization than nHAp at all stages. In contrast, in the present study, the CPP-ACP-containing formulation achieved remineralization levels statistically comparable to fluoride and nHAp at shallow depths. This discrepancy may be attributed to differences in application frequency. Whereas Vyavhare et al. applied CPP-ACP once daily, our protocol involved twice-daily 3-min applications, which may have contributed to the enhanced performance observed in this experimental setting. These findings suggest a potential dose- and frequency-dependent effect that should be considered when interpreting CPP-ACP outcomes across studies. The ΔΔS values of the nHAp- and CPP-ACP-containing formulations did not differ significantly, suggesting comparable overall remineralization despite the depth-dependent differences observed in their CSMHR profiles.

The fluoride-containing formulation showed the highest remineralization effect at 30 μm among the tested formulations but exhibited progressively lower recovery values at greater measurement depths, suggesting a predominantly surface-associated effect under the present experimental conditions. This aligns with the commonly reported surface-limited mechanism of fluoride [9,16,45]. The specific fluoride-containing phases formed during remineralization may depend on factors such as fluoride concentration, pH, and exposure duration. Previous studies have shown that conventional dentifrice-level fluoride (1450 ppm F) primarily promotes the formation of low-fluoride fluorhydroxyapatite at the enamel surface, whereas prolonged fluoride exposure favors more extensive CaF_2_ and fluorapatite formation at greater depths [61]. Given the short-duration, twice-daily application protocol used in the present study, a predominantly fluorhydroxyapatite-mediated surface reaction may plausibly explain the observed surface-associated hardness recovery. However, this interpretation remains speculative because no chemical characterization was performed. These considerations further highlight the potential relevance of adjunctive strategies when targeting deeper enamel regions.

The BAG-containing formulation displayed a different remineralization profile, with comparatively lower improvement at 30 and 50 μm but greater remineralization than fluoride-containing formulation at 100 and 150 μm. This is consistent with previous reports suggesting that the remineralization potential of BAG may be associated with the release of calcium, phosphate, and silicate ions, followed by hydroxycarbonate apatite formation [45]. These processes may help explain the hardness recovery observed at deeper assessment depths in the present study. However, the near-surface improvement in the present study was lower than that reported by Bakry et al. [62], who found strong surface recovery comparable to fluoride when BAG was kept in prolonged contact with enamel via bonding. Our findings likely reflect the shorter contact time in a dentifrice-simulation protocol. Based on previous reports, the formation of an initially amorphous HCA layer that gradually matures into a more crystalline structure may help explain the relatively lower surface hardness observed within the 14-day period [63]. These observations suggest that the performance of BAG-containing formulations may depend on exposure duration and delivery mode rather than on the intrinsic properties of the nominal active component alone. It should also be noted that commercial BAG formulations vary considerably in composition and particle characteristics. The BAG-containing dentifrice used in the present study was BioMin C, a fluoride-free formulation containing a calcium-chloride-based bioactive glass, whereas Bakry et al. [62] investigated a 45S5 sodium-calcium-phosphosilicate formulation. Previous studies have shown that different BAG compositions may exhibit distinct ion-release profiles and physicochemical behaviors, which could contribute to variations in remineralization outcomes reported across studies [46]. Therefore, differences in formulation characteristics, including composition, particle characteristics, delivery vehicles, application protocol, and contact time, should be considered when comparing findings across investigations, as these factors may influence the availability and diffusion of remineralizing ions, ultimately affecting remineralization outcomes [64].

When assessed using the integrated hardness recovery parameter (ΔΔS), the nHAp- and CPP-ACP-containing formulations demonstrated significantly greater overall integrated hardness recovery than the BAG- and fluoride-containing formulations, whereas no significant difference was observed between the BAG- and fluoride-containing formulations. The control group showed the lowest integrated hardness recovery. Although the fluoride-containing formulation demonstrated the highest recovery at the most superficial measurement depth (30 µm), its predominantly surface-associated remineralization profile may have contributed to a lower integrated hardness recovery than the biomimetic formulations. Conversely, the BAG-containing formulation showed integrated hardness recovery comparable to that of the fluoride-containing formulation despite its greater contribution at deeper assessment depths, suggesting that its lower superficial recovery offset its deeper recovery pattern within the present experimental timeframe.

The combined use of cross-sectional microhardness and SEM analysis strengthened interpretation by providing both quantitative and qualitative information. Microhardness measurements at multiple depths allowed differentiation of surface-dominant and subsurface-acting effects [30], while SEM provided qualitative visualization of cross-sectional morphological changes following treatment. Although transverse microradiography (TMR) is considered a gold-standard technique for quantifying mineral density changes [65], cross-sectional microhardness has been widely accepted as a reliable indirect indicator of enamel remineralization under controlled in vitro conditions.

SEM observations revealed that sound enamel exhibited a smooth and compact morphology with intact prism structures, whereas demineralized specimens presented irregular and porous surfaces consistent with demineralization. Following treatment, distinct morphological patterns were observed among the groups. Specimens treated with the BAG-containing formulation showed heterogeneous surface deposits; specimens treated with the CPP-ACP-containing formulation demonstrated partial occlusion of interprismatic spaces by amorphous-appearing deposits; and specimens treated with the fluoride-containing formulation exhibited surface features predominantly confined to the outer enamel surface. In contrast, specimens treated with the nHAp-containing formulation exhibited surface features consistent with a relatively continuous layer, with apparent extension into interprismatic regions. The untreated control group retained a porous morphology. Overall, these morphological findings corresponded with the microhardness results; however, SEM observations should be interpreted as qualitative support rather than direct evidence of mineral penetration depth.

Several methodological factors enhance the reliability of this study. Using sound, demineralized, and treated regions within the same tooth reduced inter-tooth variability [32]. However, because sound enamel measurements were obtained from the lingual surface whereas demineralized and treated measurements were obtained from the buccal surface, inherent anatomical differences between these surfaces may have contributed to variability in sound enamel hardness values. Human enamel was selected to maximize clinical relevance [66] and surface preparation was avoided to preserve natural lesion morphology [67]. Daily renewal of solutions ensured chemical stability throughout cycling [35], and the 14-day protocol was selected to reflect the reported time course of biomimetic systems such as CPP-ACP and nHAp and the HCA formation period for BAG [63]. It should be noted that lesion morphology is influenced by the experimental conditions; therefore, the findings should be interpreted within the framework of the applied protocol. Furthermore, the tested formulations were applied undiluted for 3 min without brushing or salivary dilution to standardize exposure across materials with different physical forms. As this protocol does not reflect routine dentifrice use, differences in formulation consistency, surface retention, and application mode may have influenced the observed remineralization outcomes, as similarly reported in previous in vitro pH-cycling studies [68], and should therefore be considered when interpreting the present findings.

As with all in vitro studies, the present findings should be interpreted with caution, as artificial caries models cannot fully reproduce the structural and biofilm-mediated complexity of natural lesions. Although pH cycling simulates demineralization–remineralization dynamics, it does not fully reproduce the oral environment, where salivary proteins, the acquired pellicle, and oral biofilm may influence ion diffusion and the behavior of remineralizing agents [69,70]. Previous studies suggest that saliva may enhance CPP-ACP localization on enamel [71], that protein adsorption around nHAp particles may affect nHAp–enamel interaction [72], and that the pH-increasing and antibacterial effects of BAG may be more relevant under biofilm-associated conditions [73]. In addition, BAG-based materials may benefit from longer surface contact times to facilitate ion release and hydroxycarbonate apatite formation; therefore, their performance under the present experimental conditions should be interpreted with caution [62]. Another limitation is that commercially available formulations rather than isolated active ingredients were evaluated. Accordingly, the observed effects should be interpreted as the performance of the tested formulations rather than the isolated effects of their nominal active ingredients. Furthermore, the tested products could not be fully standardized due to limited manufacturer information, including the particle size of the nHAp-containing formulation. Because particle size influences specific surface area and may affect remineralization behavior, this should be considered when interpreting the present findings, although nano-scale dimensions have been reported for similar nHAp particles [74,75]. Additionally, pH values of the tested formulations were not confirmed for all products, which may influence ion availability and remineralization outcomes; the pH of GC Tooth Mousse is reported as approximately 7.8. Furthermore, operator blinding was not performed during specimen preparation and treatment application, which may have introduced a potential source of bias.

Despite these limitations, the present study provides a controlled comparison of the remineralization potential of the tested commercial formulations under identical experimental conditions. The observed depth-dependent patterns may offer useful insights into their potential behavior in non-cavitated lesions; however, extrapolation to clinical conditions should be made with caution, and further in situ and clinical studies are required. Future research should employ in situ or clinical models and consider alternative delivery vehicles for BAG to maximize surface retention. Methods such as transverse microradiography may also provide more detailed information on mineral distribution. Further studies incorporating deeper and more superficial measurement points are recommended to provide a more comprehensive characterization of lesion depth.

Within the limitations of this study, all tested formulations demonstrated remineralization capacity, but their depth-dependent behavior varied. The fluoride-containing formulation predominantly enhanced the most superficial assessed region, the CPP-ACP-containing formulation showed greater superficial recovery with moderate recovery at deeper levels, the BAG-containing formulation exhibited comparatively greater recovery at deeper assessment depths, and the nHAp-containing formulation demonstrated comparatively uniform recovery across the evaluated depths within the present model. These findings may support consideration of biomimetic formulations as adjunctive options in minimally invasive caries management, particularly when subsurface remineralization is desired. Further in situ and clinical studies are needed to establish long-term efficacy and guide evidence-based application protocols.

## 5. Conclusions

Within the limitations of this in vitro study, all tested formulations promoted hardness recovery in artificially demineralized enamel. However, the extent of recovery varied according to assessment depth. The nHAp-containing formulation demonstrated the most consistent hardness recovery across all evaluated depths, whereas the CPP-ACP-containing formulation showed greater recovery in superficial regions with reduced effectiveness at increasing depths. The BAG-containing formulation exhibited comparatively greater recovery at deeper assessment depths but lower superficial recovery, while the fluoride-containing formulation produced the greatest recovery at the most superficial measurement depth, with progressively reduced recovery at deeper levels. These findings indicate distinct depth-dependent hardness-recovery profiles among the tested commercial formulations.

## Figures and Tables

**Table 1:** Composition and manufacturer information of the commercial formulations used in this study.

Commercial Formulation	Manufacturer	Composition
Biorepair	Coswell S.p.A., Funo, Bologna, Italy	Water, zinc hydroxyapatite *, glycerin, sorbitol, hydrated silica, silica, flavor, cellulose gum, tetrapotassium pyrophosphate, sodium myristoyl sarcosinate, sodium methyl cocoyl taurate, sodium saccharin, citric acid, phenoxyethanol, benzyl alcohol, sodium benzoate.
GC Tooth Mousse	GC Corp., Tokyo, Japan	Water, glycerol, CPP-ACP *, D-sorbitol, silicon dioxide, CMC-Na, propylene glycol, titanium dioxide, xylitol, phosphoric acid, guar gum, zinc oxide, sodium saccharin, ethyl p-hydroxybenzoate, magnesium oxide, butyl p-hydroxybenzoate, propyl p-hydroxybenzoate.
BioMin C	BioMin Technologies Ltd., Stoke-on-Trent, UK	Glycerin, silica, polyethylene glycol, sodium lauryl sulfate, chlorocalcium phosphosilicate *, titanium dioxide, sweetener, carbopol, potassium acesulfame.
Colgate Triple Action	Colgate-Palmolive, Guangzhou, China	Glycerin, water, zinc citrate, hydrated silica, sodium lauryl sulfate, tetrasodium pyrophosphate, arginine, sodium fluoride (1450 ppm) *, flavor, zinc oxide, cellulose gum, poloxamer 407, xanthan gum, benzyl alcohol, sodium saccharin, cocamidopropyl betaine, phosphoric acid, sucralose, CI 77891.

Abbreviations: CPP-ACP, casein phosphopeptide–amorphous calcium phosphate. * Active remineralizing ingredient.

**Table 2:** Mean (±SD) Vickers microhardness (VHN, kg/mm^2^) values of baseline, carious, and post-pH-cycling enamel at different depths for all groups.

Group	Depth	Baseline (Sound) Mean ± SD	Carious Mean ± SD	Post-pH-Cycling Mean ± SD	*p* ^1^	*p* ^2^
nHAp	30 µm	231.46 ± 29.40	144.54 ± 14.51	203.15 ± 23.44	*p* < 0.001	*p* < 0.001
	50 µm	248.85 ± 17.39	170.31 ± 10.77	225.15 ± 14.48	*p* < 0.001	*p* < 0.001
	100 µm	262.54 ± 13.15	176.08 ± 15.25	240.23 ± 13.47	*p* < 0.001	*p* < 0.001
	150 µm	250.92 ± 17.87	185.08 ± 20.57	226.92 ± 13.86	*p* < 0.001	*p* < 0.001
CPP-ACP	30 µm	249.75 ± 14.29	147.42 ± 9.76	222.92 ± 15.66	*p* < 0.001	*p* < 0.001
	50 µm	244.75 ± 12.20	163.33 ± 9.60	223.25 ± 16.59	*p* < 0.001	*p* < 0.001
	100 µm	246.17 ± 12.85	183.50 ± 11.27	216.67 ± 7.25	*p* < 0.001	*p* < 0.001
	150 µm	249.67 ± 22.56	191.92 ± 14.11	221.67 ± 14.52	*p* < 0.001	*p* < 0.001
BAG	30 µm	225.91 ± 10.14	142.75 ± 14.69	179.91 ± 10.63	*p* < 0.001	*p* < 0.001
	50 µm	230.33 ± 13.06	161.41 ± 14.10	184.25 ± 11.74	*p* < 0.001	*p* < 0.001
	100 µm	238.66 ± 17.49	163.58 ± 15.84	200.83 ± 16.53	*p* < 0.001	*p* < 0.001
	150 µm	234.66 ± 10.69	187.58 ± 14.12	210.41 ± 9.88	*p* < 0.001	*p* < 0.001
Fluoride	30 µm	228.69 ± 16.72	128.31 ± 13.73	205.15 ± 19.96	*p* < 0.001	*p* < 0.001
	50 µm	227.69 ± 15.04	151.92 ± 9.53	188.46 ± 7.56	*p* < 0.001	*p* < 0.001
	100 µm	236.00 ± 22.93	160.46 ± 12.09	177.23 ± 8.71	*p* < 0.001	*p* < 0.001
	150 µm	229.08 ± 21.68	180.38 ± 10.36	183.15 ± 9.89	*p* < 0.001	*p* < 0.001
Control	30 µm	228.62 ± 10.47	132.69 ± 12.63	137.08 ± 11.92	*p* < 0.001	*p* < 0.001
	50 µm	237.38 ± 15.82	149.92 ± 13.98	153.77 ± 12.28	*p* < 0.001	*p* < 0.001
	100 µm	238.08 ± 22.68	158.00 ± 13.31	162.15 ± 14.59	*p* < 0.001	*p* < 0.001
	150 µm	240.54 ± 24.70	161.38 ± 21.29	164.00 ± 20.16	*p* < 0.001	*p* = 0.019

*p*^1^ value for sound vs. demineralized comparison (paired *t*-test), *p*^2^ value for demineralized vs. remineralized comparison (paired *t*-test) All within-group comparisons were statistically significant (*p* < 0.05).

**Table 3:** Mean (±SD) CSMHR (%) values of enamel at different depths for all groups.

Depth (µm)	nHAp	CPP–ACP	BAG	Fluoride	Control	*p* Value
30	67.02 ± 7.35 ^b^	73.95 ± 12.44 ^b^	44.78 ± 5.51 ^a^	75.80 ± 11.74 ^b^	4.47 ± 2.54 ^c^	*p* < 0.001
50	69.31 ± 7.76 ^b^	72.63 ± 10.59 ^b^	33.05 ± 3.68 ^a^	48.29 ± 9.66 ^d^	4.46 ± 2.94 ^c^	*p* < 0.001
100	68.31 ± 4.97 ^b^	52.40 ± 8.62 ^a^	49.26 ± 6.64 ^a^	22.68 ± 7.32 ^d^	5.29 ± 3.44 ^c^	*p* < 0.001
150	64.46 ± 9.08 ^b^	52.25 ± 8.61 ^a^	48.54 ± 8.34 ^a^	5.60 ± 2.93 ^c^	3.12 ± 4.58 ^c^	*p* < 0.001

^a,b,c,d^ Different superscript letters indicate statistically significant differences among groups at the same depth (one-way ANOVA, post hoc Tamhane’s T2 test).

**Table 4:** Mean (±SD) values of the demineralization-associated integrated hardness deficit (ΔS_carious_), residual integrated hardness deficit (ΔS_post-pH-cycling_), and integrated hardness recovery (ΔΔS) for all groups. Values are expressed in VHN·µm.

Group	ΔS_carious_ (Mean ± SD)	ΔS_post-pH-cycling_ (Mean ± SD)	ΔΔS (Mean ± SD)
nHAp	9587 ± 2316	2828 ± 927 ^a^	6760 ± 1733 ^a^
CPP-ACP	8450 ± 1503	3196 ± 783 ^a^	5254 ± 1071 ^a^
BAG	8175 ± 1489	4571 ± 851 ^b^	3604 ± 733 ^b^
Fluoride	8650 ± 2041	5695 ± 1731 ^b^	2955 ± 759 ^b^
Control	10,003 ± 2186	9552 ± 2048 ^c^	452 ± 193 ^c^

ΔS_carious_ values did not differ significantly among groups (one-way ANOVA followed by Tukey’s post hoc test, *p* = 0.102). Different superscript letters indicate statistically significant differences among groups for ΔS_post-pH-cycling_ and ΔΔS values (one-way ANOVA followed by Tamhane’s T2 post hoc test, *p* < 0.05).

## Data Availability

The data presented in this study are available from the corresponding author upon reasonable request.

## Abbreviations

BAG	Bioactive glass
CPP-ACP	Casein phosphopeptide–amorphous calcium phosphate
CSMHR	Cross-sectional microhardness recovery
nHAp	Nano-hydroxyapatite
pH-cycling	pH cycle simulating demineralization and remineralization
SEM	Scanning electron microscopy
VHN	Vickers hardness number

## References

[1] Spatafora G., Li Y., He X., Cowan A., Tanner A.C.R. (2024). The Evolving Microbiome of Dental Caries. Microorganisms.

[2] Ribeiro A.A., Paster B.J. (2023). Dental caries and their microbiomes in children: What do we do now?. J. Oral Microbiol..

[3] Ludovichetti F.S., Stellini E., Zuccon A., Lucchi P., Dessupoiu N., Mazzoleni S., Parcianello R.G. (2025). Prevention of White Spot Lesions Induced by Fixed Orthodontic Therapy: A Literature Review. Dent. J..

[4] Cochrane N.J., Cai F., Huq N.L., Burrow M.F., Reynolds E.C. (2010). New approaches to enhanced remineralization of tooth enamel. J. Dent. Res..

[5] Monjarás-Ávila A.J., Hardan L., Cuevas-Suárez C.E., Alonso N.V.Z., Fernández-Barrera M., Moussa C., Jabr J., Bourgi R., Haikel Y. (2025). Systematic Review and Meta-Analysis of Remineralizing Agents: Outcomes on White Spot Lesions. Bioengineering.

[6] Prasanna Neelakantan G.H. (2014). Remineralization of the Tooth Structure—The Future of Dentistry. Int. J. PharmTech Res..

[7] Naveena Preethi P.N.C., Sakunthala B.K. (2014). Remineralizing Agent—Then and Now—An Update. Dentistry.

[8] Silva K.G., Pedrini D., Delbem A.C.B., Cannon M. (2007). Effect of pH variations in a cycling model on the properties of restorative materials. Oper. Dent..

[9] Philip N. (2019). State of the Art Enamel Remineralization Systems: The Next Frontier in Caries Management. Caries Res..

[10] Arifa M.K., Ephraim R., Rajamani T. (2019). Recent advances in dental hard tissue remineralization: A review of literature. Int. J. Clin. Pediatr. Dent..

[11] Braga R.R., Habelitz S. (2019). Current developments on enamel and dentin remineralization. Curr. Oral Health Rep..

[12] Gupta R., Kumari A.R., Sharma H., Jain L. (2017). Demystifying early carious lesion: A review. SRM J. Res. Dent. Sci..

[13] Gourismita Acharya P.A., Patri G. (2016). Recent Biomimetic Advances in Rebuilding Lost Enamel Structure. J. Int. Oral Health.

[14] Walsh T., Worthington H.V., Glenny A.M., Marinho V.C.C., Jeroncic A. (2019). Fluoride toothpastes of different concentrations for preventing dental caries. Cochrane Database Syst. Rev..

[15] Cochrane N.J., Saranathan S., Cai F., Cross K.J., Reynolds E.C. (2008). Enamel subsurface lesion remineralisation with casein phosphopeptide stabilised solutions of calcium, phosphate and fluoride. Caries Res..

[16] Buzalaf M.A.R., Pessan J.P., Honório H.M., Ten Cate J.M. (2011). Mechanisms of action of fluoride for caries control. Monogr. Oral Sci..

[17] Goldberg M. (2020). Fluorides in dental tissues: Caries prevention and fluorosis. JSM Dent..

[18] Anil A., Ibraheem W.I., Meshni A.A., Preethanath R.S., Anil S. (2022). Nano-hydroxyapatite (nHAp) in the remineralization of early dental caries: A scoping review. Int. J. Environ. Res. Public Health.

[19] Vandiver J., Dean D., Patel N., Bonfield W., Ortiz C. (2005). Nanoscale variation in surface charge of synthetic hydroxyapatite detected by chemically and spatially specific high-resolution force spectroscopy. Biomaterials.

[20] Verma P., Muthuswamy Pandian S. (2021). Bionic effects of nano hydroxyapatite dentifrice on demineralised surface of enamel post orthodontic debonding: In-vivo split mouth study. Prog. Orthod..

[21] Min J.H., Kwon H.K., Kim B.I. (2011). The addition of nano-sized hydroxyapatite to a sports drink to inhibit dental erosion: In vitro study using bovine enamel. J. Dent..

[22] Chatzidimitriou K., Theodorou K., Seremidi K., Kloukos D., Gizani S., Papaioannou W. (2025). The role of hydroxyapatite-based, fluoride-free toothpastes on the prevention and the remineralization of initial caries lesions: A systematic review and meta-analysis. J. Dent..

[23] Reynolds E.C., Cai F., Shen P., Walker G.D. (2003). Retention in plaque and remineralization of enamel lesions by various forms of calcium in a mouthrinse or sugar-free chewing gum. J. Dent. Res..

[24] Cross K.J., Huq N.L., Palamara J.E., Perich J.W., Reynolds E.C. (2005). Physicochemical characterization of casein phosphopeptide-amorphous calcium phosphate nanocomplexes. J. Biol. Chem..

[25] Sitthisettapong T., Doi T., Nishida Y., Kambara M., Phantumvanit P. (2015). Effect of CPP-ACP Paste on Enamel Carious Lesion of Primary Upper Anterior Teeth Assessed by Quantitative Light-Induced Fluorescence: A One-Year Clinical Trial. Caries Res..

[26] Santhosh V.N., Ankola A.V., Sankeshwari R.M., Varghese A.S., Ragu K., Kandasamy Parimala Y., Chavan P.J., Shankkari S. (2025). Remineralization Potential of Casein Phosphopeptide-Amorphous Calcium Phosphate, Nanohydroxyapatite Crystals, and Bioactive Glass on Initial Enamel Lesions: A Systematic Review and Meta-analysis. Int. J. Clin. Pediatr. Dent..

[27] Klarić E., Tarle A., Vukelja J., Soče M., Grego T., Janković B. (2023). Remineralization effects of Er, Cr: YSGG and/or bioactive glass on human enamel after radiotherapy—An in vitro study. Lasers Med. Sci..

[28] Drago L., Toscano M., Bottagisio M. (2018). Recent Evidence on Bioactive Glass Antimicrobial and Antibiofilm Activity: A Mini-Review. Materials.

[29] Ramadoss R., Padmanaban R., Subramanian B. (2022). Role of bioglass in enamel remineralization: Existing strategies and future prospects-A narrative review. J. Biomed. Mater. Res. B Appl. Biomater..

[30] Rodrigues E., Delbem A.C., Pedrini D., Cavassan L. (2010). Enamel remineralization by fluoride-releasing materials: Proposal of a pH-cycling model. Braz. Dent. J..

[31] Akküç S., Duruk G., Keleş A. (2023). Remineralization effect of three different agents on initial caries and erosive lesions: A micro-computed tomography and scanning electron microscopy analysis. BMC Oral Health.

[32] Hamdi K., Hamama H.H., Motawea A., Fawzy A., Mahmoud S.H. (2022). Remineralization of early enamel lesions with a novel prepared tricalcium silicate paste. Sci. Rep..

[33] Joshi C., Gohil U., Parekh V., Joshi S. (2019). Comparative Evaluation of the Remineralizing Potential of Commercially Available Agents on Artificially Demineralized Human Enamel: An In vitro Study. Contemp. Clin. Dent..

[34] Aydın B., Pamir T., Baltaci A., Orman M.N., Turk T. (2015). Effect of storage solutions on microhardness of crown enamel and dentin. Eur. J. Dent..

[35] ten Cate J.M., Duijsters P.P. (1982). Alternating demineralization and remineralization of artificial enamel lesions. Caries Res..

[36] Thierens L.A.M., Moerman S., Elst C.V., Vercruysse C., Maes P., Temmerman L., Roo N.M.C., Verbeeck R.M.H., Pauw G.A.M. (2019). The in vitro remineralizing effect of CPP-ACP and CPP-ACPF after 6 and 12 weeks on initial caries lesion. J. Appl. Oral Sci..

[37] Jefferies S.R. (2014). Advances in remineralization for early carious lesions: A comprehensive review. Compend. Contin. Educ. Dent..

[38] Reynolds E.C. (2009). Casein phosphopeptide-amorphous calcium phosphate: The scientific evidence. Adv. Dent. Res..

[39] Narayana S.S., Deepa V.K., Ahamed S., Sathish E.S., Meyappan R., Satheesh Kumar K.S. (2014). Remineralization efficiency of bioactive glass on artificially induced carious lesion an in-vitro study. J. Indian. Soc. Pedod. Prev. Dent..

[40] GC Corporation GC Tooth Mousse: Application Guide. https://www.gc.dental/australasia/sites/australasia.gc.dental/files/products/downloads/gctoothmousse/ifu/ifu-tooth-mousse.pdf.

[41] Vyavhare S., Sharma D.S., Kulkarni V.K. (2015). Effect of three different pastes on remineralization of initial enamel lesion: An in vitro study. J. Clin. Pediatr. Dent..

[42] Gutiérrez-Salazar M.d.P., Reyes-Gasga J. (2003). Microhardness and chemical composition of human tooth. Mater. Res..

[43] Ersen M.C., Çelik Z.C., Oztas M., Sahin M., Tagtekin D., Yanikoglu F. (2025). Impact of demineralization time on enamel microhardness reduction and lesion depth: An in vitro study. Cureus.

[44] Chuenarrom C., Benjakul P., Daosodsai P. (2009). Effect of indentation load and time on knoop and vickers microhardness tests for enamel and dentin. Mater. Res..

[45] Batra A., Shetty V. (2021). Non-Fluoridated Remineralising Agents--A Review of Literature. J. Evol. Med. Dent. Sci..

[46] Raszewski Z., Chojnacka K., Mikulewicz M. (2024). Investigating Bioactive-Glass-Infused Gels for Enamel Remineralization: An In Vitro Study. J. Funct. Biomater..

[47] Fernandes N.L.S., Silva J., de Sousa E.B.G., D’Alpino P.H.P., de Oliveira A.F.B., de Jong E.J., Sampaio F.C. (2022). Effectiveness of fluoride-containing toothpastes associated with different technologies to remineralize enamel after pH cycling: An in vitro study. BMC Oral Health.

[48] Almeida L.F., Marín L.M., Martínez-Mier E.A., Cury J.A. (2019). Fluoride dentifrice overcomes the lower resistance of fluorotic enamel to demineralization. Caries Res..

[49] Savas S., Kavrìk F., Kucukyìlmaz E. (2016). Evaluation of the remineralization capacity of CPP-ACP containing fluoride varnish by different quantitative methods. J. Appl. Oral. Sci..

[50] Vitiello F., Tosco V., Monterubbianesi R., Orilisi G., Gatto M.L., Sparabombe S., Memé L., Mengucci P., Putignano A., Orsini G. (2022). Remineralization efficacy of four remineralizing agents on artificial enamel lesions: SEM-EDS investigation. Materials.

[51] Sleem M., Eid E.-S.G., Abdelghany A., Farahat D. (2025). Evaluation of the remineralization potential of different bioactive glass varnishes on white spot lesions: An in vitro study. BMC Oral Health.

[52] Baskar R., Srinivasan D., Eagappan A.S., Shyamkumar V., Udhayakumar N. (2025). Comparative Evaluation of Remineralization Efficacy of a Novel Proanthocyanidin Biovarnish with Fluoride Varnish on Primary Teeth: An In Vitro Study. Int. J. Clin. Pediatr. Dent..

[53] Reguzzoni M., Carganico A., Lo Presti D., Zecca P.A., Scurati E.I., Caccia M., Levrini L. (2025). Assessment of the Effects of Enamel Remineralization After Treatment with Hydroxylapatite Active Substance: SEM Study. Appl. Sci..

[54] Li X., Wang J., Joiner A., Chang J. (2014). The remineralisation of enamel: A review of the literature. J. Dent..

[55] Arnold W.H., Dorow A., Langenhorst S., Gintner Z., Bánóczy J., Gaengler P. (2006). Effect of fluoride toothpastes on enamel demineralization. BMC Oral Health.

[56] Grohe B., Mittler S. (2021). Advanced non-fluoride approaches to dental enamel remineralization: The next level in enamel repair management. Biomater. Biosyst..

[57] Tschoppe P., Zandim D.L., Martus P., Kielbassa A.M. (2011). Enamel and dentine remineralization by nano-hydroxyapatite toothpastes. J. Dent..

[58] Swarup J.S., Rao A. (2012). Enamel surface remineralization: Using synthetic nanohydroxyapatite. Contemp. Clin. Dent..

[59] Vashisht R., Kumar A., Indira R., Srinivasan M.R., Ramachandran S. (2010). Remineralization of early enamel lesions using casein phosphopeptide amorphous calcium Phosphate: An ex-vivo study. Contemp. Clin. Dent..

[60] Somasundaram P., Vimala N., Mandke L.G. (2013). Protective potential of casein phosphopeptide amorphous calcium phosphate containing paste on enamel surfaces. J. Conserv. Dent..

[61] Lee B.-S., Chou P.-H., Chen S.-Y., Liao H.-Y., Chang C.-C. (2015). Prevention of Enamel Demineralization with a Novel Fluoride Strip: Enamel Surface Composition and Depth Profile. Sci. Rep..

[62] Bakry A.S., Marghalani H.Y., Amin O.A., Tagami J. (2014). The effect of a bioglass paste on enamel exposed to erosive challenge. J. Dent..

[63] Wang Y., Mei L., Gong L., Li J., He S., Ji Y., Sun W. (2016). Remineralization of Early Enamel Caries Lesions Using Different Bioactive Elements Containing Toothpastes: An In Vitro Study. Technol. Health Care.

[64] Elkassas D., Arafa A. (2014). Remineralizing Efficacy of Different Calcium-Phosphate and Fluoride Based Delivery Vehicles on Artificial Caries-Like Enamel Lesions. J. Dent..

[65] Bakry A.S., Abbassy M.A. (2018). Increasing the efficiency of CPP-ACP to remineralize enamel white spot lesions. J. Dent..

[66] Laurance-Young P., Bozec L., Gracia L., Rees G., Lippert F., Lynch R., Knowles J. (2011). A review of the structure of human and bovine dental hard tissues and their physicochemical behaviour in relation to erosive challenge and remineralisation. J. Dent..

[67] Xue J., Li W., Swain M.V. (2009). In vitro demineralization of human enamel natural and abraded surfaces: A micromechanical and SEM investigation. J. Dent..

[68] Sinfiteli P.d.P., Coutinho T.C.L., Oliveira P.R.A.d., Vasques W.F., Azevedo L.M., Pereira A.M.B., Tostes M.A. (2017). Effect of Fluoride Dentifrice and Casein Phosphopeptide-Amorphous Calcium Phosphate Cream with and without Fluoride in Preventing Enamel Demineralization in a pH Cyclic Study. J. Appl. Oral. Sci..

[69] Johannes N., Hertel S., Stoffel V., Hannig C., Basche S., Schmitt V., Flemming J., Hannig M. (2024). Impact of pH-Adjusted Fluoride and Stannous Solutions on the Protective Properties of the Pellicle Layer In Vitro and In Situ. Sci. Rep..

[70] Fu Y., Ekambaram M., Li K.C., Zhang Y., Cooper P.R., Mei M.L. (2024). In Vitro Models Used in Cariology Mineralisation Research—A Review of the Literature. Dent. J..

[71] Huq N.L., Myroforidis H., Cross K.J., Stanton D.P., Veith P.D., Ward B.R., Reynolds E.C. (2016). The Interactions of CPP-ACP with Saliva. Int. J. Mol. Sci..

[72] Komiyama S., Miyasaka R., Kikukawa K., Hayman R. (2019). Can nano-hydroxyapatite permeate the oral mucosa? A histological study using three-dimensional tissue models. PLoS ONE.

[73] Farjaminejad R., Farjaminejad S., Garcia-Godoy F., Jalali M. (2025). The Role of Bioactive Glasses in Caries Prevention and Enamel Remineralization. Appl. Sci..

[74] Lelli M., Putignano A., Marchetti M., Foltran I., Mangani F., Procaccini M., Roveri N., Orsini G. (2014). Remineralization and repair of enamel surface by biomimetic Zn-carbonate hydroxyapatite containing toothpaste: A comparative in vivo study. Front. Physiol..

[75] Scribante A., Dermenaki Farahani M.R., Marino G., Matera C., Rodriguez Y.B.R., Lanteri V., Butera A. (2020). Biomimetic Effect of Nano-Hydroxyapatite in Demineralized Enamel before Orthodontic Bonding of Brackets and Attachments: Visual, Adhesion Strength, and Hardness in In Vitro Tests. Biomed. Res. Int..

